# Multilayer capsules made of weak polyelectrolytes: a review on the preparation, functionalization and applications in drug delivery

**DOI:** 10.3762/bjnano.11.41

**Published:** 2020-03-27

**Authors:** Varsha Sharma, Anandhakumar Sundaramurthy

**Affiliations:** 1 Department of Biomedical Engineering, SRM Institute of Science and Technology, Kattankulathur, Tamil Nadu 603203, India; 2 SRM Research Institute, SRM Institute of Science and Technology, Kattankulathur, Tamil Nadu 603203, India; 3 Department of Physics and Nanotechnology, SRM Institute of Science and Technology, Kattankulathur, Tamil Nadu 603203, India

**Keywords:** drug delivery, functionalization, multilayer capsules, synthesis, weak polyelectrolytes

## Abstract

Multilayer capsules have been of great interest for scientists and medical communities in multidisciplinary fields of research, such as drug delivery, sensing, biomedicine, theranostics and gene therapy. The most essential attributes of a drug delivery system are considered to be multi-functionality and stimuli responsiveness against a range of external and internal stimuli. Apart from the highly explored strong polyelectrolytes, weak polyelectrolytes offer great versatility with a highly controllable architecture, unique stimuli responsiveness and easy tuning of the properties for intracellular delivery of cargo. This review describes the progress in the preparation, functionalization and applications of capsules made of weak polyelectrolytes or their combination with biopolymers. The selection of a sacrificial template for capsule formation, the driving forces involved, the encapsulation of a variety of cargo and release based on different internal and external stimuli have also been addressed. We describe recent perspectives and obstacles of weak polyelectrolyte/biopolymer systems in applications such as therapeutics, biosensing, bioimaging, bioreactors, vaccination, tissue engineering and gene delivery. This review gives an emerging outlook on the advantages and unique responsiveness of weak polyelectrolyte based systems that can enable their widespread use in potential applications.

## Review

### Introduction

**Figure 1:**
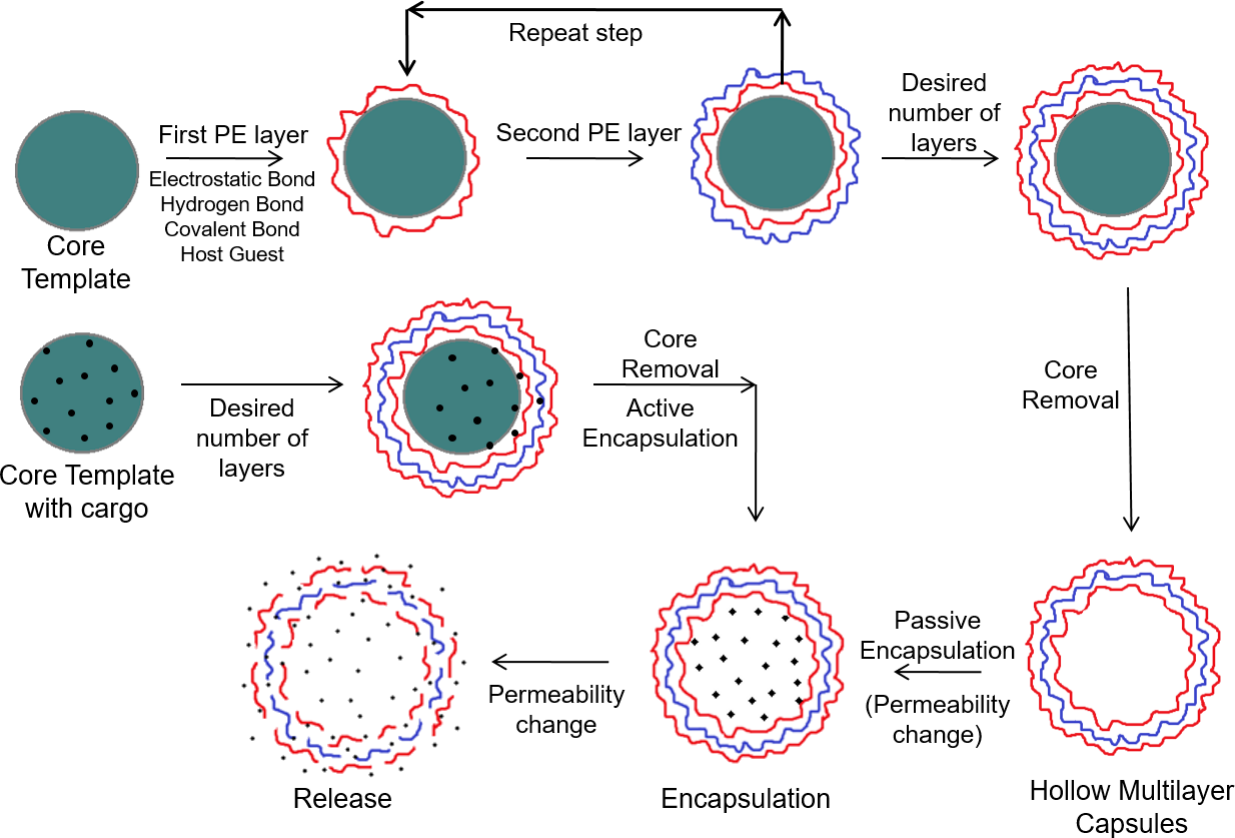
Schematic representation showing the capsule fabrication, drug encapsulation and release of loaded drug molecules.

In the last few decades, micrometer and nanometer-sized capsules made of polyelectrolytes (PEs) have been the subject of intensive research because of their significance in biotechnological and nanotechnological frontiers with applications in the fields of chemistry, physics, biology and medicine [1]. The multilayered capsules are fabricated by alternate deposition of anionic and cationic PEs on a sacrificial colloidal template, followed by the dissolution of the core. The schematic diagram of layer-by-layer (LbL) deposition on colloidal templates, core dissolution and drug encapsulation into LbL-assembled capsules is shown in Figure 1. The method of fabricating core–shell particles and multilayered hollow capsules via LbL assembly was originally proposed and developed by Iller [2] and Decher et al. [3], but it was brought into the limelight by systematic and extensive research by various research groups. The encapsulation of various macromolecules inside the hollow capsules was carried out by adjusting the physiochemical properties of the polymers in order to manipulate the shell permeability [4]. However, most of the work uses strong PEs such as polystyrene sulfonate (PSS) as one of the polymers, and thus, in order to release the payload, a disturbance in the intermolecular forces (e.g., covalent binding, hydrogen bonding and electrostatic interactions) guarding the capsule stability is necessarily required. This led to the observation of various environmental triggers such as pH, ionic strength, polarity and temperature that play a major role in manipulating the capsule permeability by modulating the shell interactive forces [5]. Following this, successful efforts were also made to release the payload under the exposure of external (modern) triggers such as laser light, ultrasound, magnetic field, enzymatic deformation and mechanical deformation. In the latter cases, the capsules were irreversibly ruptured and released the loaded molecules either in a burst or sustained manner. As weak PEs are in ionized form only in a certain range of pH or ionic strength [6], the open and closed state of multilayer capsules could be easily controlled by varying pH or ionic strength, a concept that led to widespread use in in vivo applications [7]. Much progress has also been seen in natural biopolymers such as polypeptides, polynucleotides, lipids and polysaccharides as they are biodegradable under common physiological conditions via enzymatic and pH induced degradation [8].

The advantage of LbL-assembled capsules lies in the versatility of interactions (e.g., covalent and noncovalent) between the PEs used for capsule fabrication and their nature. The multilayers showcase selective permeability by being permeable to low molecular weight compounds while impermeable to larger macromolecules [9]. The size of multilayered hollow capsules can be easily controlled by varying the size of the sacrificial template [10]. The shell thickness can also be altered by the number of layers and the preparation conditions, thus providing control over thickness and morphology. Notably, these capsules have better encapsulation efficiency (EE) and a stable zeta potential mainly due to the chemical nature of the polymers used and the assembly conditions [11]. Many studies have shown successful permeability changes for encapsulation and release via changes in pH or ionic strength for weak PE systems. The chemical nature of the polymers used along with the type of the core composition defines the mechanical properties such as elastic modulus, toughness, strength and robustness of the hollow capsules [8]. The influence of the above-mentioned properties on capsule morphology and size have been demonstrated by inducing deformations on capsules either by osmotic [12] or physical force [13] using confocal laser scanning microscopy (CLSM), atomic force microscopy (AFM), and reflection interference contrast microscopy (RICM). Fabrication conditions such as the type of polymer (e.g., thicker layers are formed by PEs having lower charge density) [14], concentration of the polymer solution (higher concentration leads to thicker walls) [14], ionic strength [15] and pH [16] also affect the types of interactions, shell thickness and permeability of the capsules.

The easier and successful encapsulation of different molecules/compounds into the capsules demonstrates the potential of multilayer capsules as drug delivery vehicles [17]. They are capable of encapsulating all kinds of substances and/or molecules ranging from enzymes, nucleic acid, peptides, proteins, therapeutic drugs, biomolecules, fluorescent molecules and nanoparticles (NPs) in their hollow cavity [18]. This can be achieved in many ways: by using the material itself as a template, by incorporating the material into core templates, or by encapsulating the drug in preformed capsules by controlling the permeability of the capsules using environmental triggers. These factors along with several introduced functionalities in the shell are also used to carry out the release of the encapsulated payload in a controlled manner. The incorporation of functionalities such as organic molecules, NPs, fluorescent dyes, polymers, nanotubes and other biomolecules into the PE multilayers during the fabrication makes it easy to control the internal structure, mechanical properties and permeability of the shell in order to induce the release of loaded cargo under exposure to external triggers.

Several reports on strong PE capsules to date show their wide use in many practical applications ranging from the loading and controlled release of therapeutic agents upon minor variations in the environmental characteristics, surface modification and suppression of inter-chain interaction to the degradation/rearrangement of LbL films under the action of physical factors [19–20]. In spite of the fact that weak PE systems can also offer many such advantages, only a few works have been reported. For instance, the responsiveness to instantaneous environmental changes (e.g., pH, ionic strength and temperature) come from the physical proximity of weakly charged groups to each other [21]. The effect of PE density can be particularly prominent when there are many charged groups present in the system. The use of different charged groups for LbL assembly can impart unique responsiveness to LbL systems as the residual charges in the multilayer films can play an important role in manipulating the polymer/polymer interactions, thus leading to easy engineering of the properties. Although recent interest has been shown in exploring weak PE system with biopolymers for several applications, their unique responsiveness and advantages are not yet addressed in detail. With the emerging applications of weak PEs, it is imperative to accurately understand their capabilities. In this review, we have summarized the work done on weak PE multilayer capsules. Their fabrication on different types of core templates, driving forces involved between different polymers, and encapsulation strategies used are discussed in detail. We also summarize the different ways of functionalization of the capsules and several internal/external triggers used to release the payload with special focus on their applications. It is believed that weak PE systems can prove to be highly efficient and suitable for several in vivo applications.

### Core templates

The most important step for hollow multilayer capsule preparation is the dissolution and complete removal of its core whose size can vary from nanometers to micrometers. The template should be inert and should not affect the chemical and mechanical properties of the polymer shell. A wide range of organic and inorganic particles, NPs, proteins, biological cells, liposomes, DNA, dyes and drugs have served as suitable sacrificial templates [22]. After serving as a support to develop multilayer assembly, the core is dissolved by using suitable solvents.

**Figure 2:**
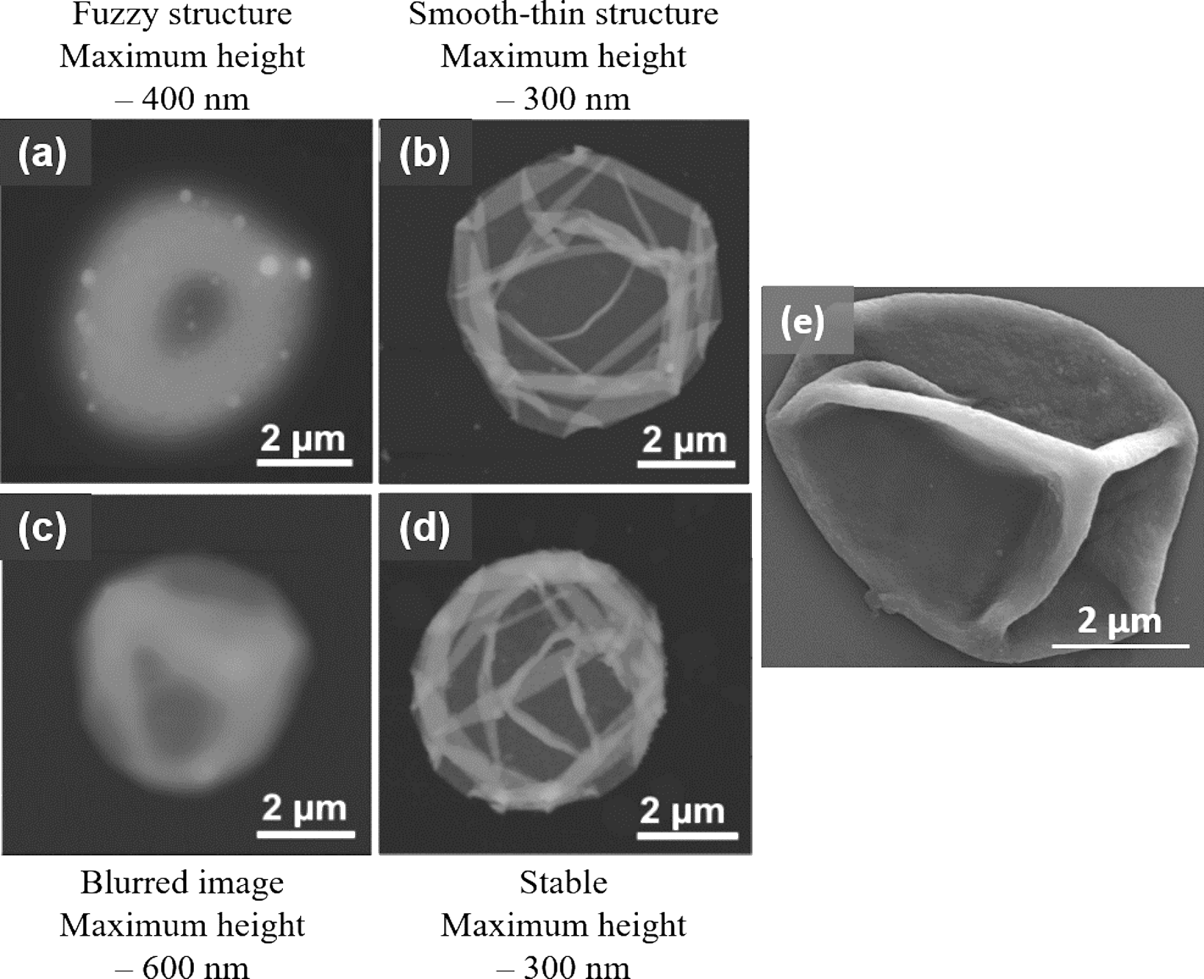
Morphological changes in PAH/PMA capsules templated on a SiO_2_ core (a–d). AFM images showing hollow capsules obtained after dissolution of SiO_2_ core deposited with (a) 8 PAH/PMA (75 KDa) layers at pH 5, (b) 8 PAH/PMA (75 KDa) layers at pH 4.5, (c) 16 PAH/PMA (75 KDa) layers at pH 4.5, (d) 8 PAH/PMA (790 KDa) layers at pH 4.5 and (e) SEM image of PAH/PMA capsule deposited on CaCO_3_ core. (Images a–d adapted and reprinted with permission from [24], copyright 2006 American Chemical Society. Image e reprinted from [29], copyright 2010 Elsevier B.V.).

Organic cores such as melamine formaldehyde (MF) and polystyrene (PS) were the few originally employed as sacrificial templates. Upon dissolution of these templates at pH < 1.6 or by organic solvents such as dimethylformamide (DMF), dimethyl sulfoxide (DMSO), hydrochloric acid (HCl) and tetrahydrofuran, the capsule showed swelling and retraction of the shell in order to diffuse out the dissolved core particles [23]. In the case of the MF template dissolution, the dissolution was incomplete as it interacted with positively charged layer components and resulted in a negatively charged complex inside the hollow capsules [24]. This was also the case with PS template removal, which also affected the shell structure limiting their biological applications [25]. The use of silicon oxide (SiO_2_) templates is quite common, however, dissolution using hazardous hydrofluoric acid (HF) limits its application. It is mostly used with strong PE systems but has also been extended to weak PE assemblies [26]. The dissolution of a SiO_2_ core in a poly(allylamine hydrochloride) (PAH)/poly(methacrylic acid) (PMA) assembly with ammonium fluoride (NH_4_F) at a suitable pH contributed to both multilayer stability and colloidal stability as shown in the AFM images in Figure 2a–d [24]. It was reported that when an 8- or 16-layer capsule core was dissolved at pH 5 and 4.5, respectively, the structure showed high mean heights suggesting trapped silica gel inside the capsules which may be due to an unsuitable pH or thick shells. However, the dissolution of the core containing 8 layers at pH 4.5 resulted in a thin and smooth capsule [27]. Similarly, biodegradable cores of polylactic acid polymer have also been investigated with several PEs, but limited by poor colloidal stability [28].

In contrast to the above-mentioned studies, inorganic templates such as carbonates are larger in size and dissolve under mild conditions at pH < 3 or by using ethylenediaminetetraacetic acid (EDTA) without affecting the PE multilayer shell. They do not form any complexes with PEs and provided intact hollow capsules after core dissolution [30]. Cores such as calcium, cadmium and manganese carbonate were successfully employed for hollow capsule fabrication [31]. Another important feature was that they can be porous or nonporous. During LbL assembly on porous cores, PE complexes were also formed in the interior and its core dissolution gave rise to a gel matrix, which helped in both protecting the capsules from high osmotic pressure and for encapsulation of macromolecules [32]. Calcium carbonate (CaCO_3_) cores have been widely used with many combinations of weak PEs to fabricate hollow capsules as in case of PAH/PMA capsules, Figure 2e [29]. More recently, hybrid CaCO_3_ templates built in with other components such as PSS, poly(styrene)-co-poly(acrylic acid) (PS-PAA) have also been reported as “functional templates” as they endowed the capsules with some special properties and enhanced stability. The PAH/PS-PAA multilayer capsules fabricated over PSS-CaCO_3_ templates retained their 3D shape even after drying and spontaneously entrapped water-soluble positively charged rhodamine B molecules similar to wet capsules [33]. PAH/PMA capsules fabricated using an alginate-doped CaCO_3_ template displayed an interconnected matrix in the interior of hollow capsules, enhancing encapsulation of cationic molecules [34]. Certain limitations such as low stability and low EE have been reported in organic and inorganic templates, respectively. Interestingly, the deposition of an additional silica layer through a biomimetic mineralization process onto a protamine/PSS microcapsule formed on a CaCO_3_ template improved the EE, storage stability and resulted in better tolerance for encapsulated enzymes against harsh environment [35]. Although none of the templates can be considered as universal, the CaCO_3_ cores have been found to be most compatible for encapsulation and release purposes.

Most of the weak PE capsules have been demonstrated with silica or carbonate cores. The crystallization of CaCO_3_ from supersaturated solutions results in porous gel-like cores which also play an important role for applications in industry and medicine [32]. Such formation depends on the experimental conditions such as pH, salt, concentration, intensity of mixing and agitation. These templates are biocompatible, easy to produce, and dissolvable by EDTA. Although crystallization processes cannot be controlled to provide uniform and homogenous cores, this matrix provides an efficient way for encapsulation. Additionally, hydrogel-based microparticles (10–200 µm) fabricated via stop flow lithography have emerged as useful templates to form custom-shaped and flexible microcapsules of poly-ʟ-lysine (PLL) [36]. The shell was formed by diffusion of PLL into an oppositely charged hydrogel matrix, enabling an easy surface modification that can be applied to a variety of PEs. Multilayer capsules formed on such a variety of cores can reveal specific characteristics and are mostly studied using microscopy techniques such as scanning electron microscopy (SEM) and transmission electron microscopy (TEM) to observe their morphological changes. Moreover, small-angle X-ray scattering (SAXS) of hollow capsules could give a detailed illustration of the inner structure and their size distribution in in situ measurements. SAXS investigations of PAH and poly-ʟ-aspartic acid multilayers deposited on poly(acrylic acid) (PAA) brush modified PS templates indicated that some PAH chains penetrated into the PAA brush [37]. Notably, SAXS proved to be a powerful tool to gain more information about the inner structure of such systems.

### Driving forces for capsule formation

**Figure 3:**
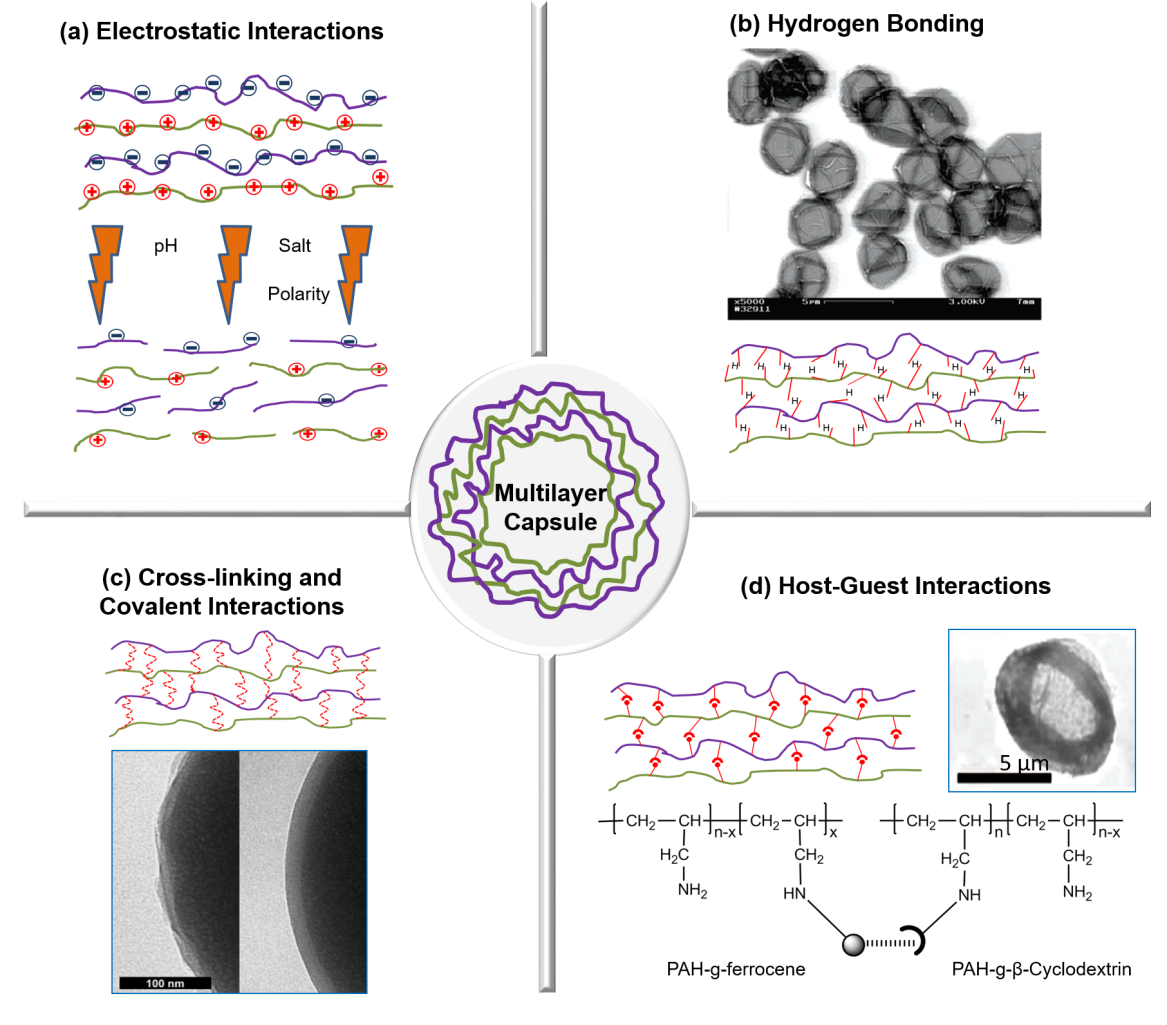
Illustration of driving forces for capsule assembly. (a) Opening of electrostatically bound multilayers upon change in physiological conditions, (b) SEM investigation of hydrogen bonded, temperature sensitive (PMA/PVCL)_5_ capsules, (c) TEM investigation of 440 nm SiO_2_ particles coated with PAH/PAA multilayers before (left) and after (right) covalent crosslinking with EDC and (d) host–guest PAH-cyclodextrin and PAH-ferrocene microcapsule assembly. Image b was adapted and reprinted with permission from [38], copyright 2005 American Chemical Society, image c was adapted and reprinted with permission from [26], copyright 2003 John Wiley and Sons, and image d was adapted and reprinted with permission from [39], copyright 2008 American Chemical Society.

The development of capsules with enhanced stimuli responsiveness, controlled loading and release is an important aspect. Different types of driving forces define the physical and chemical properties of the capsules such as permeability, stability, stimuli responsiveness and its application in vitro and in vivo. Self-assembly formed by LbL deposition of alternatively charged PEs is governed by various forces, mostly electrostatic interactions, hydrogen bonding, covalent bonding, host–guest interactions and other interactions (e.g., hydrophobic and biospecific recognition) as shown in Figure 3.

#### Electrostatic interactions

The LbL assemblies were originally applied to both charged planar substrates and colloidal particles and the main driving force for alternate deposition of oppositely charged PEs was electrostatic interaction (Figure 3a). These interactions occur in assemblies that use polycations and polyanions such as polypeptides (e.g., poly-ʟ-arginine (Parg) and PLL), polysaccharides (e.g., dextran sulfate (DS), heparin and chitosan) and various synthetic polymers (e.g., PAH, PMA and poly(ethyleneimine) (PEI)). When a negatively charged template is dipped in a solution of positively charged PE, or vice versa, a monolayer of excessively adsorbed PEs is formed that leads to the reversal of the surface charge [3]. The coated templates are then rinsed and dipped in a solution of oppositely charged polymer; again, a monolayer deposition takes place but with the restoration of the original surface charge. The rinsing step becomes important, as it would otherwise lead to complex formation and aggregation. This sequential adsorption of layers finally leads to electrostatically bound multilayers in which charge inversion is the main driving force controlling the assembly [40]. The strength and physical principles governing electrostatic interactions was recently explored by Lytle et al. by using a combination of theory, simulation and experiments on the arbitrary sequence of charges [41]. The results indicated that an increase in charge fraction and blockiness generally leads to an increase in the two-phase region of the phase diagram, thereby showing enhanced phase separation by stronger electrostatic interactions. However, the relative position of the blocks also plays a significant role in determining the phase behavior. In another work, the role and binding stoichiometry between cationic and anionic groups of PE and the effect of deposition onto existing layers was studied by using anionic fluorescent probes [42]. The amount of probe bound to an existing layer was linearly dependent on the thickness of multilayers up to 15 nm, and thereafter highly overlapped layers instead of well separated films were observed. Even when the outermost layer was negatively charged, negatively charged probes were permeable into the multilayer films but with a slower diffusion rate. The main advantage of electrostatically bound assemblies is the versatility and high susceptibility towards pH, ionic strength and polarity [43]. As studied under SEM and CLSM, the walls of PAH/PSS microcapsules become disturbed at pH < 6, forming 100 nm pores which caused fluorescein isothiocyanate-dextran (FITC-dextran) in the solution to permeate into the hollow capsule. Notably, no structural changes were observed at pH > 8 [44]. Most important was the reversibility of the capsules from open state at pH 3.5 to closed state at pH 10. The pore formation is either due to the weakening of molecular bonds through ionization or osmotic pressure developed during core dissolution. By increasing the NaCl salt concentration from 0.1 to 0.5 M, similar swelling and shrinking was observed due to the charge screening effect on multilayer bonds [44]. Similar morphological changes were observed when the temperature dependence of electrostatically bound capsules was studied with various PEs that are thermo-responsive [45]. Additionally, the effect of the dissolution of the core on the permeability of capsules was observed. At basic pH, the flow of FITC-dextran into the MF-templated capsules was immediate as compared to carbonate cores, wherein it occurred after 10 min [44]. This was explained by the fact that MF core dissolution leaves some remains inside the capsule. Upon pH change, these remains contribute to charging of wall components and affect the wall structure, thereby affecting the capsule wall integrity. In spite of the versatility, in addition to the inexpensive and easy fabrication of electrostatic assemblies, the response over a wide pH range becomes a limitation as it is biologically irrelevant. Several crosslinking methods were researched to improve the mechanical strength, lower the permeability of the walls and enhance the stability of electrostatically bound hollow capsules made of weak PEs. The electrostatic assemblies are limited to the use of charged and water soluble polymers that could lead to the problem of fouling in biological systems and decreased efficiency.

#### Hydrogen bonding

Hydrogen bonding can be used with uncharged polymers for the capsule assembly to make it responsive towards stimuli relevant to physiological conditions. The working pH range of the capsule is based on the strength of the hydrogen bond between the pair, making it suitable for specific drug delivery applications. Hydrogen bonded PAA/polyvinylpyrrolidone (PVP) multilayer films on planar surfaces were first reported by Wang et al. in 1997 [46]. It was later extended to 3D systems in 2003 wherein PVP/methyl phenol formaldehyde resin (MPR) multilayered hollow nanocapsules were obtained using SiO_2_ as a sacrificial template [47]. Notably, the excess charges induced into the multilayer films by the deprotonation of carboxylic acid groups at pH > 5 played a key role in destabilizing the hydrogen bonded films [48]. As the critical pH is closer to physiological pH, no additional changes are required to release the loaded cargo.

The fabrication of a multilayer assembly as well as its post formation manipulation is affected by molecular weight, pH, ionic strength and temperature [49]. When the critical pH is approached, the morphological changes in PVP/PMA capsules were seen with the decrease in multilayer thickness due to disruption of hydrogen bonds [50]. When the same was cross-linked by carbodiimide chemistry, the swelling was observed at higher pH, leading to the formation of highly swollen hydrogels. To induce temperature responsiveness for multilayer capsules, the hydrogen bonded assembly is more convenient in the form of polymers with lower critical solution temperature (LCST) behavior such as poly(*N*-isopropylacrylamide) (PNIPAM), poly(*N*-vinylcaprolactam) (PVCL) and poly(vinyl methyl ether) (PVME) for use as layer components [38]. The morphological changes along with the collapse of structure can be seen close to the LCST. Stable (PMA/PVCL)_5_ capsules prepared at ambient temperature and pH 2 are shown in Figure 3b. While the increase in molecular weight of the layer components led to the decrease of the film erosion rate, the effect of ionic strength was largely dependent on the type and valence state of the salts [51]. Although the hydrogen bond is much weaker than the electrostatic interactions, and could be easily damaged by the dissolution processes, their stimuli responsiveness and the ability to disintegrate at physiological pH makes them more versatile for biomedical applications.

#### Crosslinking and covalent interactions

Unlike electrostatic and hydrogen bonding interactions, the covalently linked multilayers provide a more robust way to manipulate permeability, stability and mechanical strength. Various reactions have been reported to improve the binding strength in order to increase the mechanical resistance and long term stability of the capsules. Several post fabrication treatments have also been used to strengthen the weak intermolecular interactions to fabricate stable cross-linked capsules. The PAA/poly(acrylamide) (PAAm) capsules were chemically cross-linked by water soluble 1-ethyl-3-(3-dimethylaminopropyl) carbodiimide (EDC), rendering them high stability in vitro [52]. In another work, copper ions were used to form a complex with carboxylic groups of PAA, facilitating the formation of PAA/PAH capsules via EDC crosslinking (Figure 3c) [26]. Similarly, the post crosslinking of PEI/PAA microcapsules via glutaraldehyde (GA) chemistry also resulted in better stability over a wide pH range [53]. These capsules successfully encapsulated dextran (2000 KDa) molecules without any compromise in pH responsiveness. The multilayers such as PVA/PAA and PEI/PAA could be cross-linked thermally or chemically to obtain stable hollow capsules for use in separation processes of various ionic compounds [54]. It is noteworthy that the parameters such as the nature of polycation, deposition conditions, number of polycation layers, cross-linker concentration and the nature of the dyes could significantly influence the encapsulation of anionic dyes or molecules [54].

Another method uses direct covalent chemical reactions as the driving force between comprising polymers yielding simultaneous crosslinking without the need of post treatment [55]. Stable multilayered hollow capsules of *N*-methyl-2-nitro-diphenylamine-4-diazoresin/m-methylphenol-formaldehyde resin (NDR/MPR) on a PS core based on in situ coupling were found to withstand solvent etching without further processing [55]. The reaction between epoxides and amines resulted in ultrathin, smooth and highly cross-linked structures for poly(glycidyl methylacrylate) (PGMA)/PAH hollow capsules fabricated over SiO_2_ templates [56]. These capsules were stable at extreme pH conditions (1.2–12.8) and elevated temperatures showing an elastic modulus as high as 910 MPa. By making use of the reaction between amine and aldehyde via GA chemistry, a single polymer PAH capsule can be fabricated [57]. The deposition of one PAH layer was followed by suspending the particles in GA solution to induce free aldehyde groups for deposition of the next PAH layer. A similar assembly of PEI was also reported to show the effect of the polymer molecular weight over the capsule structure [58]. When the molecular weight of the PEI was increased, the shell permeability was decreased. Notably, the covalent bonding helps in constructing single PE networks so that one polymeric component does not hinder the specific functionality of other polymeric components (e.g., biocompatibility and biodegradability). Moreover, no charge reversal process occurs, which improves the stability of multilayer capsules.

#### Host–guest interactions

Host–guest types of interactions are found within the supramolecular assemblies. Supramolecular polymers such as cyclodextrins, calixarenes, resorcinarenes and crown ethers play as host monomer units. The cup-like shape of calixerene, resorcinarenes units represents the host for their homoditopic or heterodipotic structures, i.e., two calixarene units covalently bound in various ways. Although there are many reports on calixerene [59], cyclodextrins offer a wide scope for such interactions in biological applications. They could act as a host for many biomolecules or drugs via hydrogen bonding, hydrophobic interaction or van der Waals interactions [60–63]. For instance, positively charged ferrocene-modified PAA thin films over β-cyclodextrin dimer were first fabricated in 2002, showing that LbL assemblies can be formed by overcoming electrostatic repulsion [60]. Similar attempts were made to construct LbL films by interaction between β-cyclodextrin monolayers as host and polymers modified with adamantyl groups as guests, which also resulted in stable multilayers [61]. The first stimuli-responsive supramolecular hydrogels films based on these reactions were built in 2006 using β-cyclodextrin and adamantyl modified chitosan derivatives [62]. Photo-responsive and redox-responsive hydrogels of cyclodextrin with guest molecules of azobenzene and ferrocene have also been constructed [63]. Very few attempts have been made for fabrication of weak PE capsules under this category, as shown in Figure 3d [39]. Notably, the host and guest interactions are based on their matching degree and concentration. Thus, the difficulty lies in the availability of these host and guest molecules coupled to charge repulsion between polymers.

### Encapsulation

The formulation of nanocarrier systems is effective only if sufficient encapsulation of molecules can be easily carried out. Various types of cargo such as proteins, enzymes, dyes, biomolecules, drugs and fluorescent molecules have been successfully encapsulated in hollow PE capsules by different encapsulation methods [64]. One way of encapsulation is active encapsulation, i.e., using the encapsulation material itself as a core or co-precipitation of encapsulation material within the core. Very few such studies are reported using a weak PE capsule. An anti-inflammatory drug, ibuprofen crystals of 5–40 µm in size, were encapsulated by chitosan/DS and chitosan/carboxymethyl cellulose multilayers [65]. The drug release time could be extended by increasing the crystal size and thickness of the multilayer films. Alternatively, the protein aggregates or DNA could also be used as templates to encapsulate them in PLL-succinylated PLL layers for model viral assembly or gene transfer [66].

**Figure 4:**
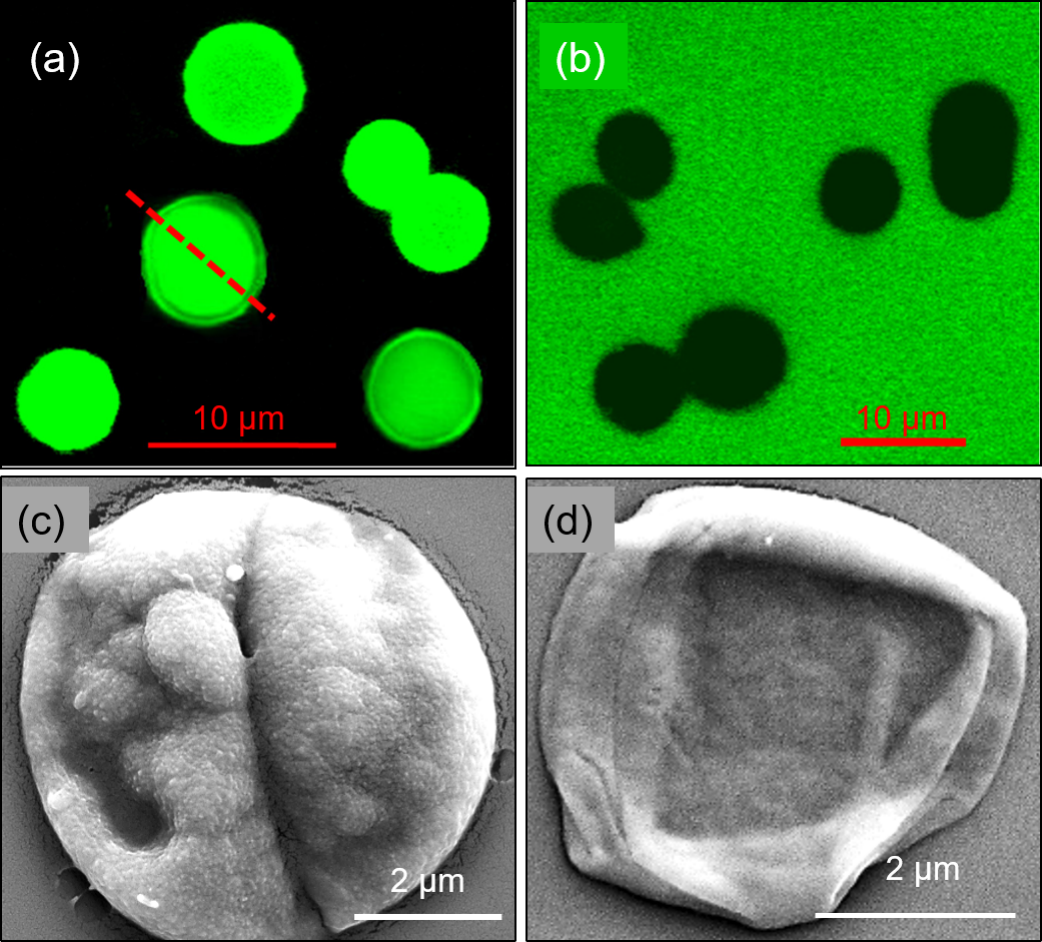
CLSM images of PAH/PMA microcapsules incubated with FITC-BSA at (a) pH 3 and (b) pH 7. SEM investigation of (c) a BSA-loaded capsule at pH 3 and (d) a capsule after the release of BSA at pH 7.4. The images in a–d were reprinted with permission from [70], copyright 2010 Elsevier B.V.

Another way of encapsulation of cargo is passive encapsulation, wherein the substances of interest are loaded into the capsules by altering the permeability of the shell using physical or chemical factors. The pH-induced encapsulation is mostly used for weak PE capsules as the changes in pH results in the generation of excessive charges. The loading of ciprofloxacin hydrochloride with an EE of 32% [29], doxorubicin (Dox) with an EE of 89% [67], enzyme-like catalase [68], horseradish peroxidase with 2.2 × 10^8^ molecules/capsule [69], and proteins like BSA with an EE of up to 65% [70] have been successfully reported in weak PE capsules by changing the open and closed state of the capsules. PAH/PMA microcapsules were successfully loaded with fluorescein isothiocyanate-bovine serum albumin (FITC-BSA) at pH < 4 [70]. Confocal microscopy was used to visualize the open and closed state of the capsules at pH 3 and 7, respectively (Figure 4a,b). Figure 4c,d shows the capsule images after encapsulation at pH 3 and after release at pH 7.4. In a similar work, the encapsulation of FITC-BSA was observed in carboxymethyl cellulose/PAH microcapsules at pH 3, which was then released at pH > 7 by manipulating the shell permeability [71]. Similar to the capsule made of strong PE, the permeability of the shell could be easily controlled by varying the ionic strength. The microcapsules underwent swelling at high salt concentrations due to the weakening of the electrostatic interactions between the PE layers by the charge screening effect [39]. The open and closed states at high and low salt concentrations were successfully demonstrated for encapsulation and release of macromolecules. The temperature-induced shrinking of the capsules was also demonstrated for the encapsulation of fluorescent model molecules [72]. The shell thickening at elevated temperatures was responsible for trapping the macromolecules inside the capsules. The hydrophobic molecules could be loaded into multilayer capsules by changing the state of the capsules from open to closed via a polarity change [73]. Alternatively, water soluble, positively charged substances were encapsulated into hollow capsules of weak PE by spontaneous as well as charge-controlled mechanisms [70,74]. The net negative charge caused by either complex formation or preloaded PE molecules in the interior of the capsule is the driving force for the encapsulation processes [75]. Another most commonly used method is the movement of cargo from lower to higher concentration via a concentration gradient based diffusion process such as in case of Dox loading in GA cross-linked (chitosan-alginate)_5_ microcapsules [76]. At low feeding concentrations (e.g., 750 µg/mL), the drug loading was attributed to the accumulation effect, whereas the overall loading was influenced by both the accumulation effect and normal diffusion processes at high feeding concentrations.

The gel-like matrix cores of inorganic CaCO_3_ and SiO_2_ provide a larger surface area for encapsulation than normal cores due to their porous nature. They can encapsulate both hydrophilic as well as hydrophobic compounds. The encapsulation of proteins in such porous structures can be done by either adsorbing model protein into core particles before PE multilayer deposition [77] or co-precipitation of protein molecules during the formation of cores [78]. The encapsulation was five times more for the latter one. After core dissolution, the capsules were encapsulated with proteins. Mostly, these studies have been demonstrated for strong PE systems. This method is not used convincingly for weak PE systems, mostly due to poor stability of the capsules and contamination issues during the core dissolution processes. Thus, despite the lower EE and drug absorption in the capsule wall rather than in the interior, pH and salt based approaches are the most widely used in weak PE systems.

### Functionalization

The surface properties of the capsule determine its permeability, which in turn influences the release profile. In order to alter the surface properties, the PE multilayer capsules can be functionalized with various organic and inorganic materials that would lead to a new way for different biological applications. These include metal NPs, magnetic NPs, click moieties, smart polymers and biomolecules such as proteins, peptides, nucleic acids, and enzymes.

#### Nanoparticle incorporation

**Figure 5:**
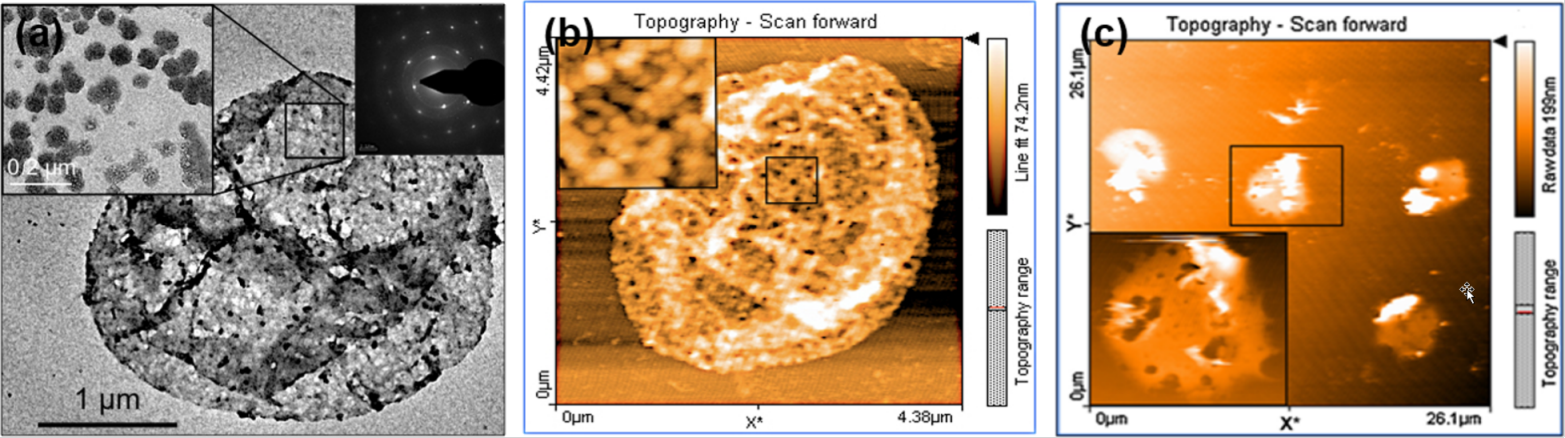
The morphological investigation of PAH/DS capsules incorporating silver NPs by (a) TEM, (b) AFM and (c) its rupture after laser irradiation. The images in a–c are reprinted with permission from [74], copyright 2011 Elsevier.

When NPs are embedded into the multilayer shell, the translocation, guiding and release of encapsulated cargo from the capsule is enabled by selectively changing the integrity or permeability. Magnetic NPs have shown greater cytotoxicity in comparison with microcapsules containing an equivalent amount of magnetite [79]. The first and foremost way of incorporating NPs into the shell is either by the adsorption of NPs over the sacrificial template or using the NP assembly itself as a template. When the sacrificial template dissolves, it truly encapsulates the NPs within the multilayers, however, the core dissolution may affect the properties of the NPs. To circumvent this limitation, preformed NPs were used as a layer component to fabricate hollow capsules incorporated with NPs. Although it resulted in stable capsules, the amount of adsorbed NPs is substantially less [80]. The third method involves the incorporation of preformed NPs into preformed hollow capsules by changing the wall permeability through several triggers [81]. Lastly, in situ synthesis of NPs in the shell itself via the polyol reduction method has proved to be effective as it results in a dense and homogenous distribution of the NPs within the capsules [74]. Figure 5a,b shows TEM and AFM images of the successful incorporation and distribution of in situ synthesized silver NPs within the PAH/DS capsule. The rupture and deformation of the capsules occurred via the formation of pores on the surface after laser irradiation at 530 nm (Figure 5c).

The encapsulation of silver, gold and iron oxide NPs has been the most common in most of the studies [80,82–84]. The incorporation of magnetic NPs (e.g., iron oxide and cobalt oxide NPs) into capsules allows them to respond to magnetic stimuli and produce heat due to magnetic energy dissipation, mechanical vibrations and motion induced in the film, thus releasing the cargo [80]. The Fe_2_O_4_-PAH capsules studied with A549 cancer cell line showed a rapid uptake, demonstrating the potential for cancer therapy. Silica and gold NPs were assembled in a one pot assembly of specifically tailored diblock polymers of PLL and poly-ʟ-cysteine [82]. The electrostatic binding between the positively charged lysine blocks and negatively charged silica NPs as well as the disulfide linkages between cysteine blocks and gold NPs resulted in two types of functionalities in the capsule. In a similar way, the capsules incorporated with noble metal NPs (e.g., gold and silver) respond to external light illumination by increased surface plasmon resonance of the outer shell electrons present in noble metals. The absorbed light is converted to heat energy, which causes layer damage, thereby opening the capsules and releasing the encapsulated material [83]. The parameters such as the preparation condition of capsules, the distribution and aggregation of NPs within the capsules, the laser power and exposure time decide the sensitivity of the capsule towards laser light induced release. For example, the presence of aggregated NPs led to the generation of more heat and thus required relatively less intense laser irradiation [84]. While the moderate intensity of radiation successfully breaks the shell and releases the loaded cargo, high intensity leads to the generation of high heat, which helps in destroying the surrounding cancer cells. Even though the advantages of NP incorporation in capsules have been immense, similar work in weak PE assemblies is still at initial stages.

#### Supramolecular functionalization

Supramolecular chemistry is the chemistry of structure, function and intermolecular bonds of supramolecular structures formed by various methods of copolymerization or binding of a substrate to molecular receptors. This can be used for specific applications such as molecular recognition, selective transport processes and the design of supramolecular devices with functional (e.g., electroactive and photoactive) components [85]. “Smart polymers” emerged from supramolecular chemistry provide reversibility of noncovalent interactions that make them responsive to external stimuli such as temperature, light and chemical environment. They might also have the capability to self-assemble into functional materials via hydrogen bonding, hydrophobic interaction, metal–ligand interaction and van der Waals forces. Among them, the major driving force of such capsules is host–guest interactions as in the case of polymers such as cyclodextrins, calixarenes, resorcinarenes and crown ethers, wherein they play as host monomer units [59,86]. Such capsules made from strong PE assemblies showed pH switchable on–off capability of the polymers without breaking their polymeric structure [87]. The pH control helped the self-assembled capsules in precipitation and dissolution by trapping of guest molecules inside. Furthermore, it led to a new strategy involving hydrogen bonding and carbamate chemistry (between carbon dioxide and amines) which resulted in supramolecular capsules that are switchable by two parameters, namely, solvent polarity and temperature [47]. Multilayer β-cyclodextrin films with ferrocene-modified PAA, adamantyl-modified dedrimer and adamantyl-modified poly(isobutene-alt-maleic acid) have been successfully reported with weak PE systems [60–61,88]. First stimuli responsive supramolecular hydrogel films based on β-cyclodextrin and adamantyl-modified chitosan derivatives have demonstrated a reversible swelling and shrinking upon changing the ionic strength [62]. At high ionic strength, the decrease in the electrostatic repulsive effect between the layers causes the compression of the assembly owing to the mass decrease and release of trapped water. The viscosity of the multilayers increased with increase in salinity. Although many hydrogels have been reported with β-cyclodextrins, only few attempts have been made in capsule formation [63]. In one successful attempt, the multiresponsive hollow capsules of PAH were fabricated by β-cyclodextrin-ferrocene reaction on sacrificial carbonate particles [39]. The swelling was progressive when the microcapsules were kept in β-cyclodextrin solution due to the gradual decrease in the degree of crosslinking. At low pH or ionic strength, the shrinking of the capsules was observed due to the alteration of charge repulsion in the PAH backbone. Moreover, this effect was reversible, rendering several applications for the controllable loading and release of cargo as demonstrated by fluorescent dextran in this study. Block copolymers such as PMA and PAAm-dimethyldiallylammonium chloride (PAAm-DMDAAC) were used to combine the electrostatic and hydrogen bonding interactions, making them stable over a broader pH range by switching the interactions between the electrostatic and hydrogen binding state [89]. While the PAAm-PMA assembly was found to be unstable at pH > 5.5 due to disruptions in hydrogen bonding, the block PAAm-DMDAAC copolymer assembly was stable even until pH 8 due to the transition from hydrogen bonding to electrostatic interactions.

#### Click chemistry

Click chemistry is the tagging of macromolecules with click moieties in order to form covalently stabilized multilayer films. This technique offers many advantages as it can be formed in aqueous as well as organic solvents. It is also applicable to a wide range of polymers, proteins, NPs and other biological molecules. The LbL click linkages have excellent physicochemical properties and are highly stable towards hydrolysis, oxidation and reduction. The different types of click reactions are as follows. First, a copper-catalyzed alkyne-azide cycloaddition (CuAAC) reaction of an azide with an alkyne catalyzed by copper is reported to form a stable triazole ring between the successive layers of the LbL assembly. Second, a strain-promoted Cu-free alkyne-azide cycloaddition (SPAAC) uses strained alkynes for reaction with azide, thereby ruling out the issues with toxic copper. A third type of click reaction, the Diels–Alder cycloaddition (DAC), is a reversible reaction between different types of diene and dienophile resulting in thermo-responsive products. A fourth type, the thiol-ene reaction, is a reaction between thiol and alkene groups in the presence of a radical source as catalyst. The radicals can be generated through light, thermal or a redox initiated approach. The most studied is the azide-alkyne cycloaddition reaction due to the high regioselectivity and quantitative transformation with no side reactions. The detailed description of these methods can be found in a recent review by Such et al. [90].

The first application in 2006 utilized CuAAc for LbL assembly of PAA functionalized by alkyne (PAA-Alk) and azide (PAA-Az) in the presence of copper to form covalent PAA films which were stable over a broad pH range [91]. This method was later extended to various other polymer films such as combinations of PAA-PAH-poly(*N*-hydroxypropylmethacrylamide) (PHPMA) [92]. The first hollow capsules based on CuAAc method was formed by azide and alkyne modified PAA along with rhodamine dye over a silica template. The hollow capsules exhibited a pH responsive behavior with reversible swelling and shrinking at acidic and basic pH [93]. In a separate study, the poly(ethylene glycol) (PEG)-based post-functionalization of pH-responsive click capsules of biodegradable PLL and poly(ʟ-glutamic acid) (PGA) rendered their low fouling capability against specific protein binding [94]. Hydrogen bonded films and hollow capsules of alkyne-modified PVP and PMA were stabilized with a bifunctional azide crosslinker containing disulfide bonds [95]. The exposure of these capsules to pH 7.2 led to the breaking of hydrogen bonds and complete removal of PMA, resulting in stable PVP capsules with intracellularly degradable disulfide linkages. These multilayers were also found to be low-fouling to various proteins and negligibly cytotoxic. In a related work, the PEG-based films and capsules fabricated with redox responsive moieties via disulfide linkage showed high stability in biological buffers, low toxicity to human cells and low fouling characteristics [96]. Similar low-fouling hybrid capsules with a controlled degradation profile were prepared by combining two different systems of PVP_Alk_-PMA and PGA_Alk_-PVP to form stratified PGA_Alk_-PVP_Alk_ capsules [97]. The degradation could be controlled by the number as well as position of the non-degradable PVP_Alk_ layer. The drug-loaded PVP_Alk_ capsules were even shown to overcome multidrug resistance with Dox and paclitaxel against LIM1899 colorectal cancer cells [98]. A new kind of dual (pH and redox) responsive, charge shifting click capsules based on poly(2-diisopropylaminoethyl methacrylate) (PDPA) were also fabricated by a similar method [99]. These capsules were stable and showed 50% size reduction at physiological pH (7.4), however, under stimulated intracellular pH they swelled up to 120% by degradation of disulfide bonds. A rapid and efficient release could be obtained by the synergistic effect of dual stimuli.

Other click chemistries like thiol-ene have also been reported because of their light-initiated and metal-catalyst-free approach. PVP along with PMA functionalized by thiol (PMA_Thiol_) and ene (PMA_Ene_) groups were used to fabricate hydrogen bonded hollow capsules via UV crosslinking [100]. It was further demonstrated that these capsules can be incorporated with functionalized PEG_Ene_ to induce reactive and low fouling properties. Notably, the SPAAC and DAC approaches have not been widely used for synthesis of capsules. The use of the click approach highly depends on the type of application. For instance, the use of UV light in the thiol-ene approach limits its application in drug delivery as it can damage DNA and cross-react with cysteine residues in proteins. The DAC approach may also affect cysteine residues in proteins that might undergo Michael addition to result in malemides [90]. Similarly, the use of copper in CuAAC may be undesirable in certain biological applications as it is toxic to cells. This copper content can also cause aggregation of proteins and degradation of some biomolecules, which can be avoided by using chelating agents [101].

#### Incorporation of biomolecules

Biomolecular ligands such as proteins (e.g., antibodies, peptides, receptor molecules, etc.), lipids and carbohydrates have long been used for targeted delivery. The attachment of these ligands to the external multilayers can be achieved by covalent as well as noncovalent interactions. For example, the HuA33 antibody specific to A33 antigens expressed on colorectal cancer cells were adsorbed on capsules from a buffer at pH 7.4 [102]. Both electrostatic and hydrogen bonding contributed to the binding of antibodies to the capsule external layer and to retaining their immunological activity. As mentioned in the previous section, the antibodies were used to functionalize PVP_Alk_ low fouling capsules using covalent click chemistry to target cancer cells [101]. They were very effective against LIM2405 colorectal cancer cells even at a low concentration of 0.1% cancer cells in the total cell population. Protein A (obtained from Staphylococcus aureus) was adsorbed on PGA/PLL film via electrostatic attraction and the in vitro cell contact with this protein mainly occurred via local film degradation [103]. The antitumoral, antitoxic, anticarcenogenic, antifungal and antiparasitic properties of protein A could be a good application in implants or tissue engineering. Another strategy for biomolecular functionalization is covalently linking the receptor specific ligands to one of the layer components that are known to interact with cancer cell receptors. For instance, the improved cell adhesion and proliferation was observed in multilayer films such as PGA/PLL, PAH/PAAm and hyaluronic acid (HA)/chitosan when one of the polymeric pair is grafted with an arginine–glycine–aspartic acid (RGD) peptide sequence [104–106]. Similarly, the PLL/PGA film showed better cell attachment when plain PGA was replaced with a laminin 5 peptide grafted PGA [107].

The homogeneous adsorption of charged lipids such as dipalmitoyldiphosphatidic acid (DPPA), dipalmitoyldiphosphatidylcholine (DPPC) and sphingosine over a capsule surface has been achieved in two ways: 1) the adsorption of lipid vesicles via electrostatic interactions and 2) the solvent exchange mechanism wherein lipid molecules dissolved in organic solvents were slowly exchanged with water [108]. The addition of the lipid layer not only provided long term stability but also slowed down the rate of permeation of small molecules through the capsule wall, making them efficient carriers for controlled release. The later work on capsule/lipid systems incorporated with neoglycolipid or folate-linked lipid showed high affinity to lectin (concanavalin A) and breast cancer cells (MCF-7) [109]. The efficient delivery of the daunorubicin hydrochloride (DNR) anticancer drug to cancer cells was achieved through folate-targeted sodium alginate/chitosan capsules. The lipid coating on the PAH/PSS microcapsule surface significantly reduced the permeability of the capsule walls [110]. Alternatively, carbohydrate functionalization has been widely used in hepatic drug delivery systems. For instance, the capsules incorporating a galactose branched polymer (PGEDMC) as one of the PE have shown specific recognition abilities with peanut agglutinin (PNA) lectin rather than nonspecific concanavalin A [111]. Although carbohydrate-related functionalization has been mostly explored for strong PE systems, DNA functionalization was also reported for thiol-functionalized PMA-PVP multilayer capsules [112]. Thus, the capsules functionalized with biomolecules have shown better performance for both sustained and targeted drug delivery applications [113].

#### Carbon compounds

**Figure 6:**
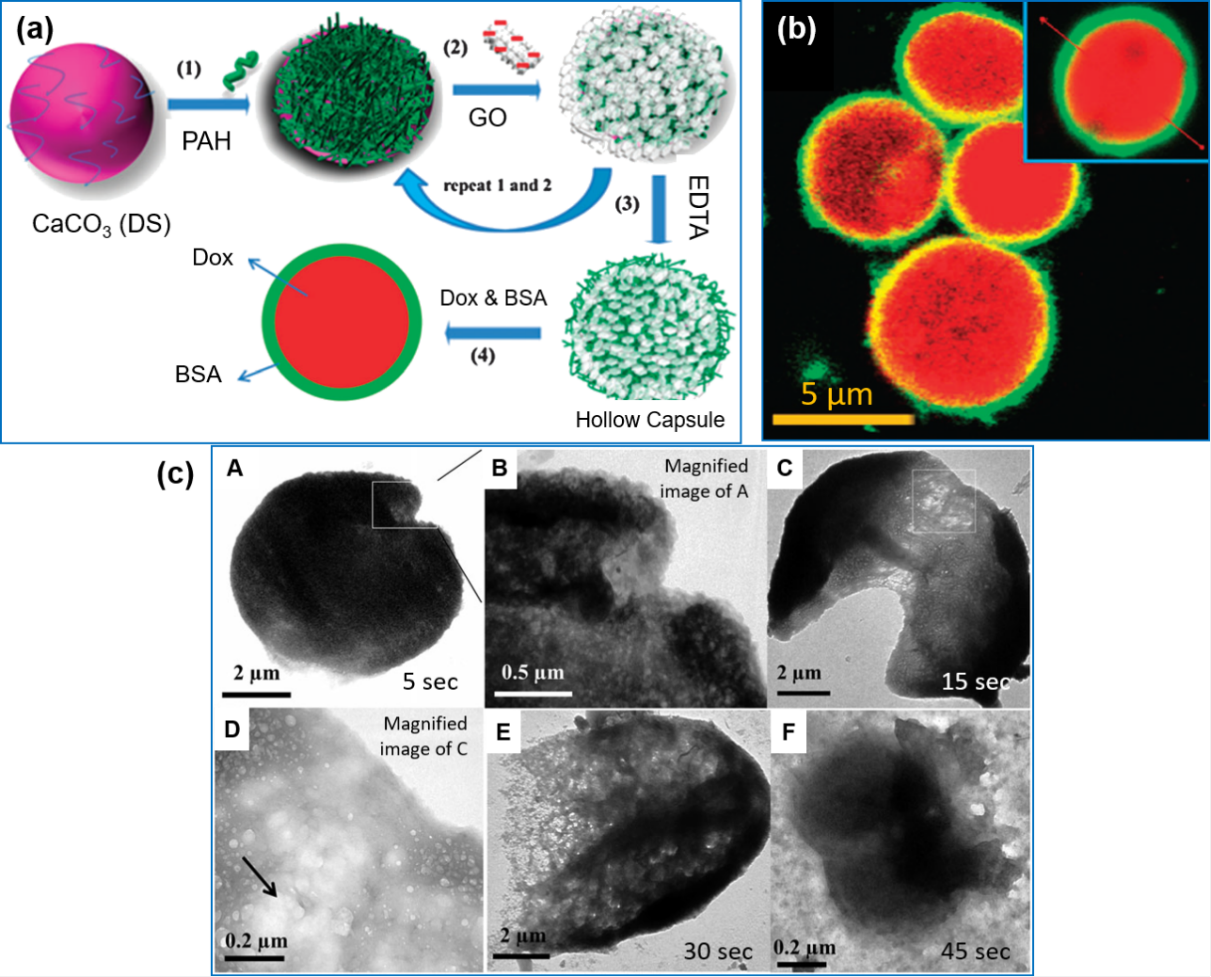
(a) Illustration of the fabrication of PAH/GO microcapsules, (b) CLSM investigation showing the encapsulation of Dox and FITC-BSA in PAH/GO capsules and (c) the response of the GO/PAH capsules to laser light at 1064 nm over an exposure time of 5 to 45 s. The images in a,b were republished with permission from [117], copyright 2012 Royal Society of Chemistry and the image in c was republished with permission from [118], copyright 2013 Royal Society of Chemistry.

Several other components have been utilized to functionalize capsules in many ways. Hollow capsules of approximately 400 nm in size with negligible toxicity to cells were reported by the oxidative self-polymerization of dopamine solution onto silica particles [114]. The thickness of multilayer films is comparable to the LbL multistep technique and can be easily controlled by varying the polymerization time. This single-step technique resulted in a wall thickness similar to the LbL multistep technique and was applicable to other particles of different size and porosity. Because of the broad absorption and interesting mechanical properties, carbon-based materials were also reported for the functionalization of hollow capsules. When the microcapsules were embedded with carbon nanotubes (CNTs) in the shell, the rigidity of the shell was improved upon drying and resulted in freestanding structures. The capsules modified with CNTs ruptured upon laser light irradiation [115]. The introduction of graphene oxide (GO) nanosheets with PDDA as multilayers caused the migration and rearrangement of chains compared to PDDA/PAA multilayers [116]. The PDDA/GO multilayers showed improved resistance to damage and maintained a defect-free surface even after several post treatments with NaClO/NaOH/HCl solutions. It is worth noting that GO/PAH multilayer microcapsules showed a unique permeability when compared to conventional capsules and provide the option to encapsulate multiple drugs by simple incubation (Figure 6a) [117]. Notably, FITC-BSA and Dox were adsorbed onto the GO layer through multiple interactions (e.g., electrostatic interactions, π–π stacking forces, hydrophobic and hydrogen bonding) between oxygen functional groups of GO and nitrogen/oxygen groups of BSA (Figure 6b). The near-infrared (NIR) responsiveness of the system as a function of time was also later explored for externally controlled drug delivery as shown in Figure 6c [118]. As the irradiation time was increased from 5 to 45 s, the pore formation and expansion of the capsules was seen followed by complete rupture at 45 s.

### Triggers for drug release

Key requirements for drug delivery vehicles is the successful release of the encapsulated drug at the diseased site for effective action in a controlled manner. The chemical and physical properties of the capsule can be easily altered by the shell constituents and its thickness. The PE membranes are mostly permeable to low molecular weight dyes and ions but are impermeable to high molecular weight molecules. In addition, the functionalization of the membrane provides a means to alter the permeability and imparts stimuli responsiveness. Internal stimuli include chemical or biological stimulus encountered internally such as pH, ionic strength, polarity, temperature, enzyme function and receptor recognition. The external stimulus, on the other hand, is the interaction with externally applied fields such as a magnetic field, ultrasound, laser light, or mechanical stress.

#### Internal triggers

**pH-responsive systems:** The human body and other tissues exhibit variations in pH (e.g., stomach pH 1–2, intestine pH 8.4, endosomal pH 6–6.5, normal tissues pH > 7.4 and cancerous tissues pH < 6.8) which makes pH sensitive systems interesting for drug delivery. As the change in external pH affects the interaction of the PE complex, the release could be triggered by targeting different parts of the body. When the pH is altered close to the p*K*_a_ value, the protonation/deprotonation of the PE occurs and leads to the swelling of the capsules by generation/reduction of residual charges in the multilayer films [119]. Notably, the charge density of weak PEs decreases at the p*K*_a_ value, making it less charged and leading to decreased multilayer stability due to weaker interactions between the layer components. The most studied pair in multilayer capsules is PAH/PSS wherein PAH provides the pH responsiveness to the system. As discussed in encapsulation studies, the PAH/PSS capsules displayed an open state at pH < 6 leading to the release of encapsulated FITC-dextran [120] and FITC-albumin [44]. It is important to note that the nature of the core and its dissolution process also significantly affects the pH response of the capsule. Notably, the oligomers left after the dissolution of MF templates affect the capsule permeability, especially for low molecular weight cargo, by increasing the osmotic pressure inside [44]. The molecular weight of the polymer and the number of layers also has a significant effect on the pH response [25]. The pH-responsive PAH/PMA capsules fabricated via crosslinking were found to be stable over a wide pH range of 2.5–11.5 and showed reversible swelling between pH 2.7–2.6 [121]. At low pH, the hydrophobic forces of PMA counteracted the weaker electrostatic attractions between the layer components and stabilized the capsules. Using the same balance of electrostatic and counteracting hydrophobic interactions, the PVP/PMA capsules exhibited pH sensitivity in both acidic and basic solutions [122]. Biodegradable capsules of chitosan/PGA with unique acidic pH responsiveness were reported by Imoto et al. [123]. While the encapsulated FITC-dextran was completely released at pH 1, the release was lower at neutral and alkaline pH. In a different work, the biocompatible and biodegradable alginate/chitosan capsules templated on liposomes showed pH responsiveness based on the end layer, i.e., the end layer effect [124]. Notably, the capsules with chitosan as the outer layer were stable at acidic pH while the capsules with alginate as the outer layer were stable at all pH values between 4.6 and 8. The methods such as GA-mediated covalent LbL crosslinking [53,58] and click chemistry [93] have also resulted in pH-tunable capsules showing impermeability over a specific pH range while being permeable at other pH values. Furthermore, these methods can be applied to a range of polymers, enzymes and proteins. The cyclodextrins-modified dextran supramolecular capsules encapsulated with Dox via host–guest interactions have demonstrated the tumor-specific release of Dox [125]. While the capsules were found to be stable at physiological pH, they burst released the loaded drug and inhibited growth of HeLa cells at acidic pH of cancerous tissues.

**Ionic strength:** The swelling and shrinking of microcapsules has been established upon exposure to different salt concentrations. Unlike the pH-based approach, the permeability change is not restricted to weak PEs but can be effectively used for strong PE systems as well. The salt ions screen the electrostatic interactions between charged polymers, leading either to the softening of the layer structure due to weakened electrostatic attractions or to the formation the pores in the multilayer network [126]. The permeability coefficient exhibits a non-linear dependence on the salt concentration. It is worth noting that the high concentration of NaCl could be used to prevent the dissolution of the poly(ethylene oxide) (PEO)/PMA film at high pH by reducing the electrostatic repulsions among the ionized groups of hydrogen bonded layers [48]. With the same phenomenon, the hydroxypropylcellulose (HPC)-PAA capsule size is decreased as a function of increasing salt concentration [127]. The effect of a wide range of NaCl concentrations from 5 to 500 mM has been studied to show that the ionic strength influences the range as well as the amplitude of electrostatic forces [128]. The capsule shrinking is largely dependent on the absolute concentration of the salt at equilibrium state and the nature of the salt. Notably, the strongest effect is observed for weakly hydrated anions [129]. The PMA hydrogel capsules also showed a gradual decrease in diameter by 1.6 times with increasing salt concentration [130]. The successful release of encapsulated FITC-dextran was achieved at a high salt concentration of 600 mM.

**Polarity:** Organic solvents have been used to alter the permeability of polymer capsules to encapsulate molecules of interest inside the hollow capsules. By changing the polarity of the solvent, the urease enzyme was easily encapsulated in PSS/PAH microcapsules [131]. The capsules demonstrated a closed state in water and an open state in ethanol. Such opening and closing of capsules is reversible and can be used for the release of the encapsulated drug. The environmental triggers are advantageous for their reversible properties and can be experimented with weak PE systems as well.

#### External triggers

**Magnetic field:** As human tissues are transparent to magnetic fields, the functionalization of capsules with magnetic NPs allows targeted delivery under an externally applied magnetic field. The first ever magnetic multilayer shell was formed by integration of iron oxide in poly(diallyldimethylammonium chloride) (PDADMAC)-PAH multilayers adsorbed on PS latex beads [132]. The same technique was extended to form the first hollow magnetically responsive capsules by removal of the core and incorporating magnetic NPs in the shell [44,133]. The uptake of capsules by cells can be drastically increased by exposure to magnetic fields. When PAH/PSS capsules functionalized with magnetic NPs and fluorescent NPs were injected into a flow channel system cultured with breast cancer cells, the accumulation of capsules was observed at the edge of the applied magnetic field [44]. It resulted in an increased local concentration and drastic internalization of capsules into cells at the edge as compared to places where the magnetic field was absent. Along with the targeting of capsules at a specific location, the magnetic field can also lead to shell rupture by inducing magnetic energy dissipation and mechanical vibration of NPs in the capsules. The high frequency magnetic field (HFMF) of 50–100 kHz directly caused heat generation and conformational changes in iron-oxide-coated PAH/PSS microcapsules due to the motion of the NPs [80]. The enhanced release of entrapped Dox was observed through the formation of nanocavities in the PAH capsule shell. This heat generation might even result in complete rupture of capsules depending on the external magnetic field and duration of the applied stimulus. These capsules also showed high intake by A5495 cancerous cell lines. Both the heating and stress on the PE shell due to magnetic NP alignment was responsible for the increased permeability in the capsules. Similar magnetoresponsive microcapsules were prepared with an additional lipid bilayer coating, in which the magnetic stimuli resulted in a phase transition of the lipid membrane due to heating of the NPs and release of fluorescent calcein without rupturing the capsules [134]. Another way is to combine both magnetic and metal NPs, wherein the magnetic NPs take care of targeting and the vibrations caused in the metal NPs work to distort the layer, making it extremely permeable to macromolecules like FITC-dextran [135]. Ferromagnetic gold-coated cobalt (Co@Au) NPs embedded inside PAH/PSS walls were observed to rotate when an external alternating magnetic field of 100–300 Hz was applied, which subsequently disturbed and distorted the capsule wall and drastically increased its permeability to FITC-labeled dextran [136]. The release rate of entrapped drug could be controlled by the strength of the magnetic field, duration of exposure, and the amount of NPs deposited onto the capsule wall [137]. These characteristics make them a suitable carrier for in vivo drug delivery.

**Laser light irradiation:** Once the capsules have reached the targeted site, the heat generation by various light stimuli can be used for destruction of the shell and releasing the encapsulated molecules. When capsules incorporated with metal NPs, light-sensitive polymers or photodynamic therapy (PDT) molecules are exposed to an external light stimulus, the heat generation at the interphase of the NP/polymer causes the rupture and change in permeability. The use of anisotropic gold/silver NPs as light absorbing moieties within the capsule provides an advantage of absorption in the NIR region (700–1400 nm), a spectral region that has high transmission and low absorption by biological tissues [137]. When exposed to NIR light of 1064 nm and 10 ns pulse, the PSS/PAH capsules incorporated with gold NPs showed 850% increase in the release of encapsulated FITC-dextran [138]. Notably, there was no significant release from the control capsules. While a lipid (dilauroylphosphatidylethanolamine) layer coating on the capsule shell helps in preventing drug leakage before release, the capsules functionalized with monoclonal IgG antibodies could facilitate the delivery to a target site. It is to be noted that in situ synthesis of metal NPs is much more efficient for such applications as they spread homogenously [139]. This was carried out by a polyol reduction method in PAH/DS capsules. The capsules showed a significant increase in the permeability under laser light exposure at 530 nm due to creation of nanometer sized pores and finally resulting in complete rupture of the capsules. The rupture of capsules is largely dependent on parameters such as NP size, their distribution, laser energy and time of exposure. It is worth noting that the moderate exposure of light can also help in the cellular uptake of the capsules and release cargo into the cytosol of cells, however, high exposures may also lead to cell death [140]. While IR laser light at high intensity can destroy capsules, the capsules could be used for bioimaging by exposing them to lower intensity light [83]. The microwave illumination of polymer capsules containing NPs showed that the molecular vibrations occurred by electromagnetic polarization with minimal heat generation. The parameters such as the frequency, power of radiation and presence of NPs controlled the extent of the deformation of the capsules [83]. Upon exposure to microwaves (2.5 GHz, 100 mW), the gold NP incorporated capsules showed burst release in about 4 min, whereas, the capsules without NPs took about 10 min to break the capsule wall, which can be useful in sustained drug release.

The release of cargo by ultraviolet (UV) radiation has also been studied in many systems [141]. UV radiation causes chemical changes in the PEs showing pronounced shrinkage in PE containing aromatic groups and negligible shrinkage in PE without aromatic groups [142]. In 2010, Koo et al. fabricated capsules with walls containing photoacid generators (PAGs). These PAGs decreased the pH of the solution by releasing protons upon UV radiation, which in turn caused swelling of the capsules [143]. The opening and closing of the capsules was thus obtained by alternate UV exposure and washing with neutral water. Azobenzene-modified polymers have also shown conformational changes in the entire chain upon UV exposure [144]. In a recent study, hematite NPs of gadolium oxide (doped with europium and terbium) were used as templates for PAH/PSS multilayers and radioluminescence-based Dox release was demonstrated [145]. Here, radioluminescence offers the advantage of greater tissue penetration and thus helps to image thick tissues. These capsules showed enhanced release at pH 5, and are thus more effective for cancer treatment. Their paramagnetic properties also make them potential MRI contrast agents. However, such light-triggered systems may also be harmful to human cells as in the case of UV-based approaches. UV-based methods have limited penetration in living tissues and also might damage the normal cells. Compared to this, NIR-triggered systems are more suitable as they have deeper penetration into living cells, can be easily focused and have no harmful effects on normal cells. Radio waves have deeper penetration but they are difficult to focus and hence cannot be used for deep tissue.

**Ultrasound:** The use of ultrasound waves in various hospital equipment prove their efficiency to be used for release purposes both in vitro and in vivo without affecting healthy tissues. The release is activated due to the occurrence of cavitations in fluids when ultrasonic waves with a frequency more than 20 kHz are used. As the waves pass through the sonic probe, microbubbles of air are formed that start to oscillate in the surrounding fluid and finally collapse causing cavitation and formation of enormous energy in the fluid. This induces shear forces in the capsule layer leading to its disruption [146]. On applying ultrasonic waves, the capsule shell is torn into pieces leading to the release of the drug. The presence of NPs make the capsules mechanically more stable during shorter treatment times [147]. On increasing the ultrasonic power (100 to 500 W) on PAH/PSS capsules embedded with iron oxide NPs, the capsules break into small pieces of about 2–16 µM in size due to the increase in the size of the cavitation bubbles [148]. Notably, the capsules without NPs were only deformed after sonication. For a homogenous distribution of NPs, in situ synthesis of silver NPs in PAH/DS capsules has also been reported [149]. The burst release of FITC-dextran within 5 s was observed upon exposure to ultrasonic irradiation of 170 W and 50 Hz. Kolesnikova and co-workers explained that the varying concentration of zinc oxide NPs in the capsule shell can increase the sensitivity of the capsules to ultrasound [150]. They presented a theory according to which the change in volume fraction of zinc oxide NPs can control the mechanical properties by decreasing the Young’s modulus and shell elasticity while increasing the fragility and sensitivity to ultrasound.

The core material also influences the deformation upon ultrasonic treatment. The capsules formed over inorganic CaCO_3_ templates became fully ruptured [147] while the capsules formed over organic templates (e.g., PS and MF) were only deformed [148,150] after 10 s of ultrasound treatment at 20–100 W. The insoluble complexes observed in the interior of the capsules played an important role in the rupturing process. Mostly, an ultrasound frequency of >1 MHz has been used in medicine as it has deeper penetration with minimal side effects. The rupture of capsules at low power causes the capsules to disintegrate to an extent enough to cause drug release. However, the use of high power results in the unnecessary destruction of the entire capsules. Further, the shell properties can be tuned to achieve better sensitivity to ultrasonic waves. Owing to its vast use in present applications, the use of ultrasound as an external trigger has great potential to be explored.

**Temperature:** The most developed approach for thermally induced release from capsules is by introducing temperature responsive polymer derivatives of poly(*N*-isopropylacrylamide) (PNIPAM). Above its LCST, PNIPAM chains undergo a phase transition from a coiled to a globule state, making hydrophobic connections with surrounding polymer chains. The PNIPAM-based systems are extensively investigated for temperature-induced release processes as the LCST temperature is closer to physiological temperature. The PNIPAM-PAH microgel thin films fabricated over a glass substrate exhibited temperature-induced release of insulin and Dox [151]. When multilayer capsules of PNIPAM/PAA were subjected to a temperature higher than its LCST, the permeability of PNIPAM was suppressed which was otherwise reversible in nature [152]. Also, the hydrogen bonded thermoresponsive PNIPAM/PAA multilayer films showed reversible encapsulation and release behavior when the temperature is changed [153]. The most adapted way to fabricate temperature-responsive microcapsules is to use PNIPAM block copolymers, including short anionic or cationic chains. Notably, the capsules fabricated by cationic and anionic PNIPAM block copolymers showed considerable decrease in size and permeability at elevated temperatures due to structural rearrangements in the shell [154]. However, the thermoresponsive behavior of the capsules was limited as this process was only partially reversible. Recently, poly(2-oxazoline)/tannic acid (TA) based multilayer capsules have shown stability over a wide range of pH and demonstrated the ability to release loaded bioactive molecules at a physiological temperature of 37 °C [155–157]. The tuning of the temperature responsiveness by addition of salts has also been reported [158]. The LCST temperature decreased upon addition of salts causing precipitation of PNIPAM chains inside the capsules. The configurational transition of PNIPAM from a globule to a coiled state occurred upon cooling the capsules below LCST. As this phenomenon is reversible, the temperature responsiveness becomes repeatable and reversible with retained thermosensitivity. By using poly(*N*,*N*-dimethylaminoethyl methacrylate) (PDMAEMA) as one of the layer components, the temperature and pH (i.e., dual) responsive microcapsules were fabricated via LbL assembly [159]. When the pH was increased, the capsules shrunk, showing the transition from open to closed state at a narrow pH range of 7–8. Similarly, at an elevated temperature of 60 °C, capsule shrinkage of about 54% was observed, thus allowing easy loading with high efficiency. Notably, the increased ionic strength resulted in increased permeability due to salt-induced PE rearrangements.

### Applications

Easy fabrication, efficient encapsulation and the ability to alter the properties of LbL-assembled PE capsules, make them useful in various areas ranging from drug delivery, imaging, sensing, tissue engineering and medicine [160–161]. Additionally, the possibility to introduce different functionalities on the capsule surface provides external control over the capsule’s release properties. Biocompatible polymers and crosslinking of weak PEs have been used to improve the stability in various biological applications.

#### Therapeutics

**Figure 7:**
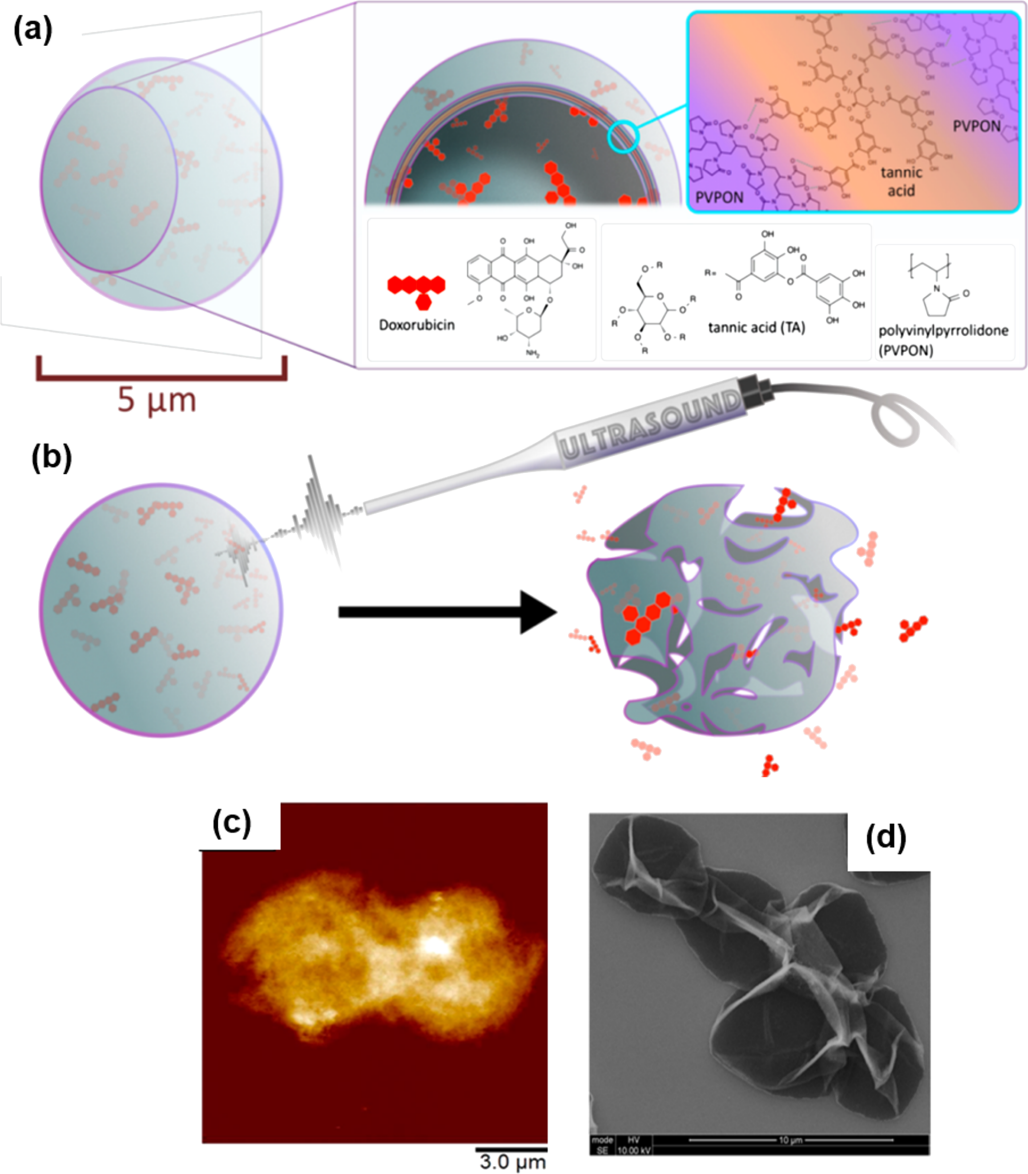
Schematic illustration of (a) the formation of PVP/TA capsules loaded with Dox, (b) the ultrasound triggered release of Dox, along with images from a morphological investigation of the capsules by (c) AFM and (d) SEM. The images in a–d were adapted and reprinted with permission from [163], copyright 2017 American Chemical Society.

Multifunctional carrier systems have been reported to effectively deliver drugs into the target cells or tissues [8]. Along with hydrophilic drugs, even hydrophobic drugs have been efficiently encapsulated by different methods. The cellular uptake of capsules loaded with hydrophobic drugs for PDT demonstrated their use as anticancer agents [76]. The coating of capsules with low fouling polymers such as PEG provides protection from various degrading proteins and the body’s phagocytic system [94]. Chitosan-DS nanocapsules loaded with ciprofloxacin or ceftriaxone were proposed as effective therapeutics against the intraphagosomal pathogen *Salmonella* [162]. In vitro and in vivo experiments showed effective clearance of the infection at a dosage significantly lower than free antibiotics due to the increased retention time of ciprofloxacin in blood and organs when it was delivered by capsules. Theranostic applications were recently observed by ultrasonically irradiating the capsules made of PVP and TA in order to deliver Dox as shown in the schematics in Figure 7a,b [163]. AFM and SEM images of the fabricated capsules are also shown in Figure 7c,d. The release of loaded molecules could be easily changed from sustained to burst profile by increasing the power of ultrasonic irradiation.

#### Anticancer therapy

The successful release of chemotherapeutic drugs from click capsules made of weak PEs has been demonstrated for intracellular environmental triggers such as pH [164]. Notably, microcapsules functionalized with monoclonal antibodies showed better specificity and cellular uptake during the in vitro experiments to target colorectal cancer cells [101–102]. In vivo experiments of chitosan/alginate multilayered capsules in mice models demonstrated the successful release of loaded Dox into acidic cancer cells [76]. The direct injection of drug-loaded capsules into the xenograft tumor of a mouse showed a sustained release of anticancer drug that led to reduced tumor growth. It is worth noting that the drug-loaded microcapsules showed better tumor regression than free drug [165].

Cancer cells are heterogeneous in many aspects, such as, some may have an elevated glutathione (GSH) level (leading to their acidic nature) or some may produce extra reactive oxygen species (ROS). A tumor may be at any stage or even these stages may coexist in different regions of one tumor. Capsules responding to one particular stimulus may release the drug only in one region of the tumor, thereby proving to be less effective. Thus Wang et al. reported capsules using a self-assembled amphiphilic chemotherapeutic pro-drug of thioester linked SN38 that responded to both GSH and ROS by releasing the parent drug SN38 via thiolysis [166]. The nanocapsules (≈100 nm in diameter) were successfully able to target tumor cells by enhanced permeability and retention (EPR) effect. Recently, it has been demonstrated that a microcapsule system fabricated over calcium chalcogenide NIPAM nanocrystals released the loaded Dox under NIR light irradiation [167]. The local temperature increase of laser light irradiation made the thermoresponsive capsule shrink and release the loaded Dox molecules. In vitro and in vivo chemotherapeutic results indicated their potential in chemotherapy and photothermal therapy.

#### Vaccination

Self-exploding capsules with a semipermeable multilayer shell made over a microgel core offer excellent carriers in viscous environments such as mucus. The encapsulated cargo is given strong propulsion into the environment and can travel longer distances in a shorter time unlike other capsules from which release occur at a slower rate by Brownian diffusion [168]. Such microcapsules were designed to be vaccine delivery systems and a single injection may include different microcapsules, exploding at different time intervals. PMA hydrogel capsules have been used for DNA encapsulation and oligopeptide delivery to white blood cells in vitro and in vivo. Ovalbumin encapsulated in PMA hydrogen bonded capsules were shown to enhance antigen presentation to CD4 T-cells [169], whereas the same encapsulated in DS/Parg electrostatic capsules enhanced antigen presentation to CD8 T-cells [170]. These systems provided fast and efficient induction of immune response in mice experiments. Immunocompatibility studies with peripheral blood mononuclear cells stated no occurrence of apoptotic activity when incubated with different multilayer capsules having either a positively or negatively charged outer surface [171].

#### Medical imaging

LbL capsules have proved to be promising candidates as imaging probes for magnetic resonance imaging (MRI) by incorporating either paramagnetic metal–ligand complexes (mostly gadolinium ligands) conjugated with polymer or superparamagnetic iron oxide NPs [172]. Magnetically responsive nanocapsules with the option for both pH-triggered drug release and MRI imaging were prepared recently by PLL/alginate multilayer deposition over silica-coated iron cores, delivering Dox and diethylenetriaminepentaacetic acid gadolinium(III) dihydrogen (Gd-DTPA) to MCF-7 breast cancer cells [173]. The release rate of Gd-DTPA, an MRI contrast agent, was much slower as compared to Dox showing its enhanced stability and advantage in multimodal MRI tracking of the magnetic capsules and the drug. Multifunctional, water soluble and biocompatible MRI contrast agents were fabricated by assembling a PLL/PGA cross-linked shell (via EDC coupling) with superparamagnetic iron oxide NPs followed by assembly with G5.NH_2_-FI-FA dendrimers [174]. By using folic acid (FA) as a targeting ligand for cancer cells and fluorescein isothiocyanate (FI) dye as an imaging probe, a multilayer capsule could be effectively used for MRI imaging of cancer cells. Reported by Shi et al., this was the first example of a successful in vivo MRI study in mice which showed a significant decrease in tumor signal intensity within 24 h [174]. Biodegradable trifunctional cross-linked PMA capsules with size of 300 nm were fabricated for co-encapsulation of Dox and perfluorohexance (PHF) for use in ultrasound imaging and ultrasound-induced drug delivery [175]. The in vitro cell assay showed the ability of these capsules to enter the cytoplasm of tumor cells via the EPR effect and the intracellular delivery of Dox. Notably, the use of PHF enhanced the imaging signal through acoustic droplet vaporization. More recently, multifunctional microcapsules incorporated with either single-walled carbon nanotubes or gold NPs on the surface have been reported for both NIR-induced release and photoacoustic imaging [176]. They served as a very good absorber in the NIR region and provided strong enhancement of photoacoustic imaging modality in both water and blood. Parg/DS microcapsules functionalized with Fe_3_O_4_ NPs have proved to be effective magnetic resonance contrast agents [177]. Low frequency alternating magnetic fields have also proven effective in inducing subtle changes in doxycycline release from PAH/PSS/Fe_3_O_4_ microcapsules without inflicting damage to the cells after 30 min of exposure [178]. Furthermore, doxycycline delivered by magnetic microcapsules enabled site specific delivery and local function using a static magnetic field, while non-targeted sites remained unaffected.

#### Biosensors

Capsules containing one or more weak PEs can be directly used as pH sensors because intracellular pH plays an important role in most of the cellular events. By linking pH sensitive seminaphtho-rhodafluor-1-dye (SNARF) with layer components (e.g., dextran), the multilayer capsules can be used as a pH sensor [179]. Notably, the functionalized capsules emitted red fluorescence in alkaline conditions while emitting green fluorescence after entering into the cells due to the acidic environment in cell compartments. By monitoring the color changes, the location of ingested capsules in the lysosomal/endosomal compartments of the cell could be visualized. Polymer capsules with ratiometric ion sensitivity towards ions such as protons, sodium, potassium and chloride have also been described at the single capsule level by conjugating different probes and indicators to dextran molecules [180]. The fluorescence response was measured by titration fluorimetry and fluorescence microscopy. It is worth noting that the detection of several ions in parallel can be tricky due to emission overlap, cross talk of indicators to non-target analytes and interference with sample pH. To overcome these problems, bar-coded capsules were proposed wherein each capsule was externally tagged by a unique luminescent code [181]. This was done by functionalizing the outermost layer with quantum dot codes of different sizes in appropriate ratios while encapsulating the enzyme and indicators in the inner core. Proton sensitive multilayer capsules with encapsulated ion fluorophores were taken up by cells and resided in lysosomes for days [182]. Upon cellular stimulation with pH-active agents, the real time measurements revealed the kinetics and mechanisms involved for intracellular pH changes with respect to different agents.

The successful encapsulation of enzymes in capsules provides protection against the degradation on the one hand while providing the option to tune the permeability of loaded enzymes at the required rate for enzymatic reactions in sensor applications on the other hand. As substrates and reaction products can easily diffuse through the membrane, it can be used for continuous reactions. Microcapsules incorporated with a fluorescence resonance energy transfer (FRET) couple could be used as a continuous glucose sensor. The competitive replacement of one of the partners in a FRET couple led to a decrease in the fluorescence that could be correlated to the amount of analyte [183]. Chinnayelka and McShane [183] used dextran and apo-glucose oxidase (high affinity to β-ᴅ-glucose) as the FRET couple to detect glucose. Glucose easily permeated through the capsule membrane, replaced apo-glucose oxidase and decreased the fluorescence intensity. The decrease in the intensity of fluorescence is a function of the glucose concentration present in the sample. This type of optical biosensor, termed as “smart tattoo”, has been reported for the encapsulation and release of anti-inflammatory drugs over a long period of 4 weeks [184]. The anti-inflammatory drug was observed to reduce the inflammation at the implant site in vivo. Various capsule-based metabolite sensors for urea, cholesterol and glucose, and even a single capsule sensing system for lactate and oxygen have also been fabricated recently [185].

#### Bioreactors

**Figure 8:**
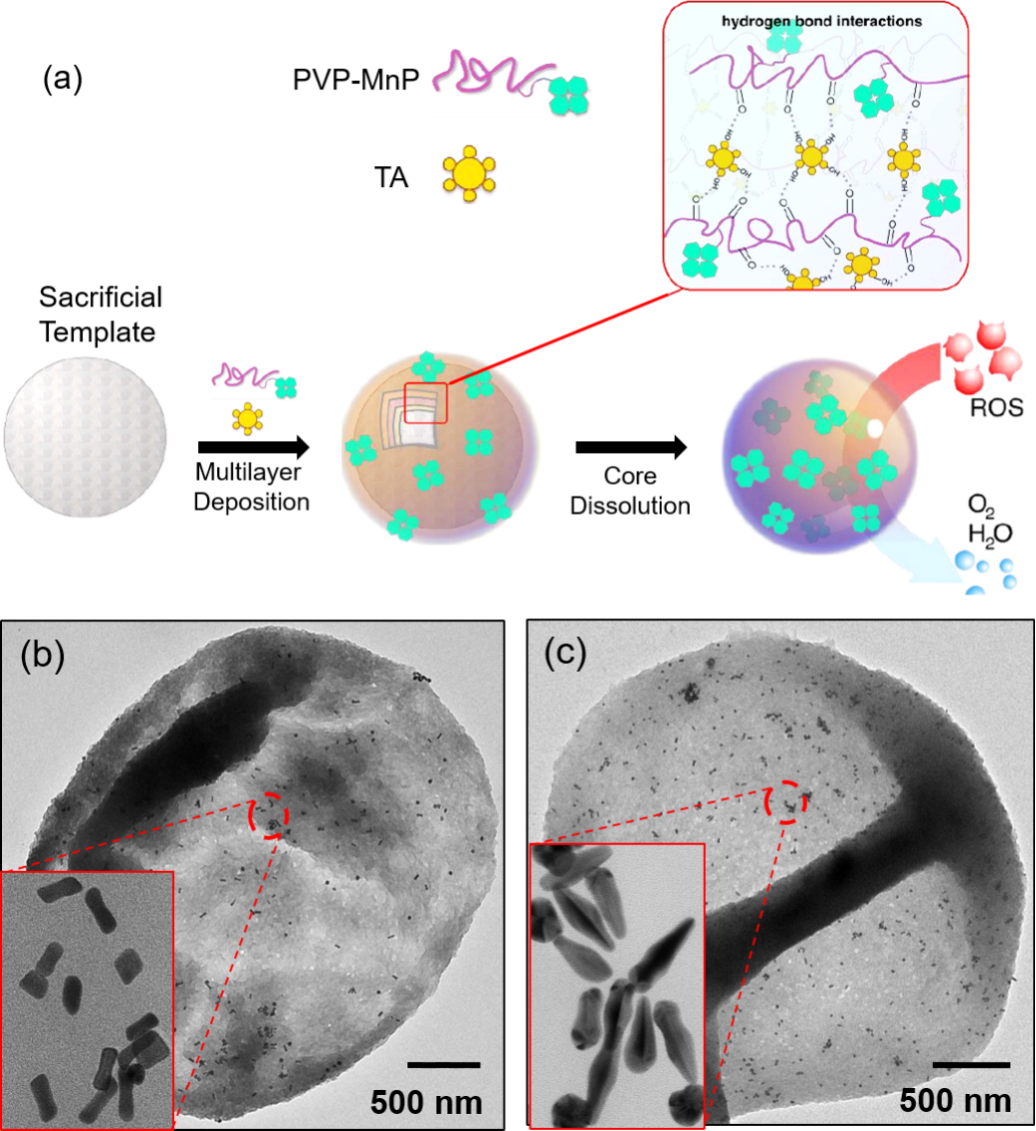
(a) Schematic representation showing the assembly of MnP-PVP/TA multilayers on silica template to obtain a hollow capsule for ROS scavenging. TEM investigation of PAH/DS microcapsules incorporating (b) gold nanorods and (c) gold bipyramids for catalysis application. The image in a was adapted and reprinted with permission from [189], copyright 2017 American Chemical Society and the images in b,c were adapted and reprinted with permission from [190], copyright 2019 American Chemical Society. Further permissions related to the material excerpted should be directed to the American Chemical Society.

Capsules having the ability to accommodate active biomolecules in the large cavity have found application in the biomedical field as microreactors, cell mimics and artificial organelles [186–187]. A wide variety of enzymatic reactions have been performed in the polymer capsules. The PAH/DS/TA microcapsules exhibited an excellent scavenging capacity for hydrogen peroxide and hydroxyl radical, suggesting better antioxidant properties [188]. Catalase enzyme encapsulated in the capsules was used to prevent oxidative stress in an in vitro inflammation model depicting that the PE shell can make encapsulated enzymes more stable compared to free enzymes. The capsules made of PVP copolymer containing a manganoporphyrin modality (MnP-PVP) and TA can mimic enzymatic antioxidant superoxide dismutase-like and catalase-like activity for efficient free radical scavenging [189]. The schematic illustration of the formation of the capsules is depicted in Figure 8a. The inclusion of a MnP-PVP layer as an outer layer enhanced radical scavenging as compared to localization of a MnP-PVP layer either in the middle or inner part of the capsule shell. Another system of PAH/DS incorporating gold anisotropic nanorods was investigated for continuous reduction reaction of *p*-nitrophenol to *p*-aminophenol [190]. TEM images of gold nanorods and gold bipyramids incorporated in PAH/DS microcapsules are shown in Figure 8b,c. Thus, these capsules have high potential as microreactors in the field of catalysis as well.

#### Tissue engineering and artificial cells

Tissue engineering requires production of the extracellular matrix (ECM) from stem cells which is difficult to achieve in vitro. The encapsulation of stem cells into PLL/HA multilayers provides a suitable environment for better interaction of stem cells and growth and instructional molecules to achieve desired matrices [191]. The subcompartmentalization or fusion of capsules represent a major step towards the fabrication of artificial cells [192]. The multilayers have also proved to be efficient in vascular therapies for the development of suitable grafts for repairing damaged vessels or arteries [193].

#### Delivery of nucleic acids

Polymer capsules for the delivery of genes and siRNA to the cell nucleus or cytoplasm have been proven to be efficient in protecting them from denaturation in endo-lysosome, oxidizing bloodstream, and extracellular environments [194]. Cross-linked PMA hydrogel and PMA/PLL capsules were used for successful encapsulation and release of siRNA in a reduced environment to target the cancer-related anti-apoptotic factor survinin [195]. DS/Parg microcapsules have also been reported as safe, efficient and non-viral delivery systems for the delivery of CRISPR-Cas9, a gene editing tool for the treatment of various genetic diseases [196]. The internalization of capsules labeled with cyanine-7 into HEK293T-d tomato cells was studied by CLSM and 3D reconstruction images. In addition to low toxicity and high internalization, the capsules could also be degraded to release the cargo into the cell microenvironment, indicating high potential for gene delivery.

## Conclusion

Over the past few decades, multilayer capsules have proved their ability in the fields of therapy, medicine, sensing and genetics. In this review, we have summarized the recent progress in the use of weak PEs for the fabrication of multilayer capsules. Their unique responsiveness to internal/external stimuli and easy manipulation of properties makes them efficient candidates for various applications. The advantages and disadvantages of driving forces involved in the formation of multilayers and encapsulation of cargo is also described. Notably, the crosslinking between the polymer layers have resulted in stable systems, however at the expense of stimuli responsiveness. The capsules modified with metal/magnetic NPs or biomolecules could be easily targeted with external stimuli for the release of encapsulated cargo. Recent progress in the use of multilayer capsules for therapy, biosensing, bioreactors, and gene therapy applications paves the way for their widespread use. Thus, exploring the use of such weak PE systems can prove to have high potential in in vivo applications.
